# Protocol to culture *Leishmania* protozoans for lipophosphoglycan extraction and purification

**DOI:** 10.1016/j.xpro.2024.103468

**Published:** 2024-12-04

**Authors:** Lisa Ulrike Teufel, Jéssica Cristina dos Santos

**Affiliations:** 1 Department of Internal Medicine and Radboudumc Center for Infectious Diseases (RCI), Radboud University Medical Center, Nijmegen, the Netherlands; 2 Department of Medical BioSciences, Research Institute for Medical Innovation, Radboud University Medical Center, Nijmegen, the Netherlands

**Keywords:** Cell Biology, Cancer, Immunology, Metabolism, Microbiology

## Abstract

Lipophosphoglycan (LPG) is a macromolecule on the surface of *Leishmania* spp. parasites. The biochemical structure of LPG varies throughout the parasites’ life cycle between proliferative (procyclic) and infective (metacyclic) stages, as well as between species and strains. Here, we outline a protocol for growing *Leishmania* parasites *in vitro* to harvest LPG. We describe steps for parasite differentiation and LPG extraction and purification. LPG has applications in medical research, such as in trained immunity and immunotherapy for cancer.

For complete details on the use and execution of this protocol, please refer to dos Santos et al.^1^

## Highlights

• Protocol for growing *Leishmania* parasites *in vitro*
• Steps for starting, maintaining, differentiating, and freezing parasites
• Optimized for LPG extraction and purification without radioactive labeling

## Abstract (editor highlights)

Publisher’s note: Undertaking any experimental protocol requires adherence to local institutional guidelines for laboratory safety and ethics.

## Graphical abstract

**Figure:**
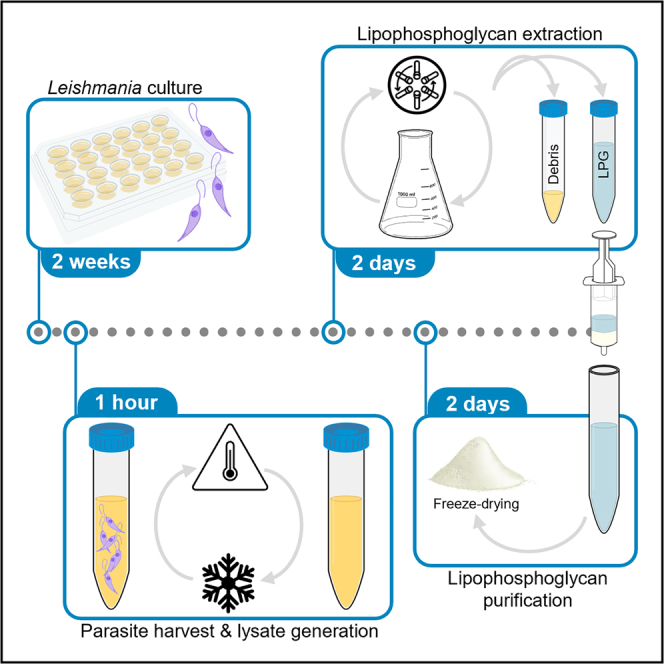

## Before you begin

Leishmaniasis is a collective term for several tropical diseases caused by protozoans of the species *Leishmania*. Clinical manifestations in humans range from localized self-healing skin lesions to chronic disease which affect large areas of the skin, mucosa, and/or inner organs. Disease outcome largely depends on the infecting *Leishmania* species and/or strain as well as on host-factors. Although millions of infections occur annually, treatment options are still suboptimal, also because they differ depending on the infecting species and the immunological status of the host, and no vaccine is available.^2^

Some *Leishmania* surface antigens possess immunogenic properties which promote disease establishment, mediate immune invasion, and thus contribute to disease progression. These antigens, such as the macromolecule LPG, are highly species-, strain-, and life cycle-specific.^3^ Therefore, they can be used to aid a fast diagnosis to choose the correct treatment regimen. However, they could also be exploited for vaccine development or antigen-specific immunotherapy approaches. Symptoms of therapy-unresponsive patients with cutaneous leishmaniasis and diffuse cutaneous leishmaniasis have been successfully treated with immunotherapy combining the tuberculosis vaccine Bacillus Calmette-Guérin with heat-killed *L. amazonensis* promastigotes or with standard therapy and lysates of *L. amazonensis* and *L. braziliensis* promastigotes.^4^^,^^5^ Moreover, we have investigated the highly immunogenic LPG of *L. braziliensis* as inducer of trained immunity with potent anti-cancer properties in mice.^1^

In-depth, structural characterization of LPG is therefore an important step in improving healthcare for this tropical, neglected disease. The protocol presented below describes specifically the extraction of metacyclic *L. braziliensis* LPG from clinical isolates maintained as laboratory culture. However, the protocol is suitable for different *Leishmania* spp. and life cycle stages. We have also used these procedures for extraction of metacyclic LPG from *L. amazonensis* and *L. chagasi*, as well as for procyclic promastigote LPG of *L. braziliensis*. All steps can be performed in a laboratory without microbiological safety level, as the parasites depend on the sandfly vector for successful infection.

### Optimize culture medium

**Timing: Approximately 2 weeks**

This section describes how to determine whether the growth medium suits the *Leishmania* species/strain of interest before starting the production of large-scale cultures for LPG extraction.

**CRITICAL:** Certain *Leishmania* spp. are sensitive to the availability of nutrients in fetal bovine serum (FBS) or its composition. As FBS is a natural product, batches have to be tested to determine whether the species of interest reaches the exponential growth phase needed to generate sufficient parasite numbers for differentiation and harvest.

***Note:*** Once a culture has been established, we advise to test multiple batches of FBS in a well growing, FBS-sensitive such as *L. braziliensis* and to store aliquots at −20°C.

1. Prepare a small batch of *Leishmania* culture medium, and note FBS company and LOT-number.

***Note:*** A different LOT-number from the same company is considered a different FBS stock.

2. Thaw procyclic parasites from starter culture (10 × 10^6^) in 37°C.
3. Add 6 mL medium to a 15 mL falcon tube and add the cells drop-wisely while continuously gently mixing.
4. Centrifuge for 10 min at 3000 × *g* at 21°C.
5. Resuspend the parasites in 1 mL medium and seed to a 24-well polystyrene cell culture plate (1 mL/well).
6. Culture the parasites at 26°C in a non-shaking incubator for 2 days.

***Note:*** Close the plate with plastic tape to avoid evaporation of the culture medium.

**Figure 1:**
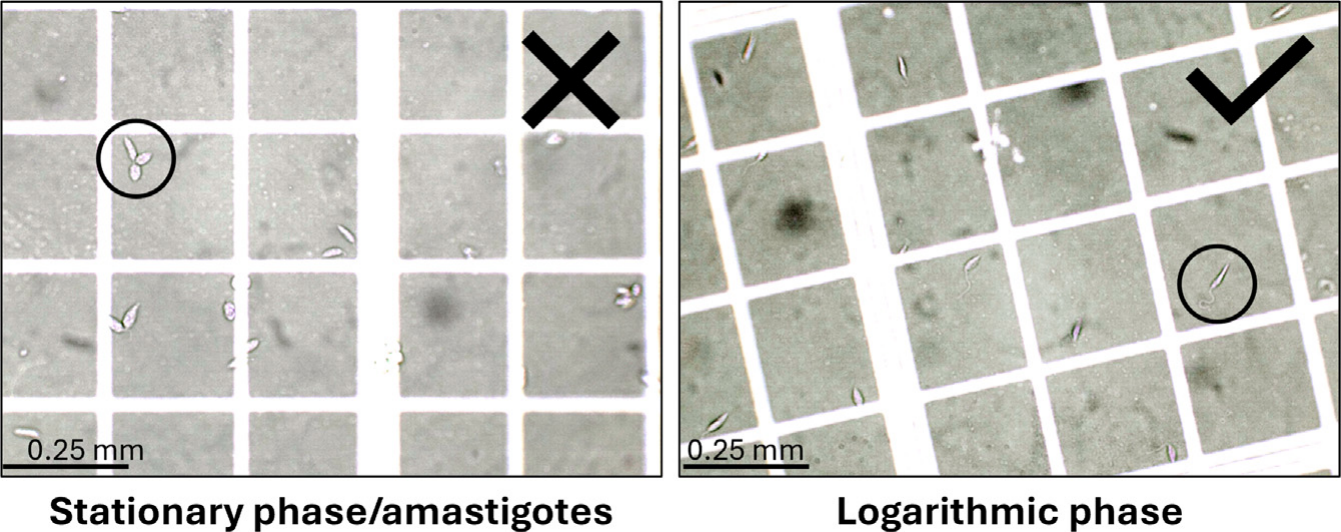
Light microscopic images of immobilized stationary and logarithmic phase promastigotes in a counting chamber (Left) rounded stationary phase promastigotes or non-flagellated amastigotes; (right) flagellated logarithmic phase promastigotes 10× magnified.

7. Check culture microscopically in the culture plate to determine whether parasites have adjusted to the medium and grow exponentially.
  ***Note:*** Adjusted parasites will be motile and form rafts (Methods video S1) (problem 1), and the majority of the culture is in the insect stage (flagellated form). Movement of the flagellum is visible under an inverted light microscope using phase-contrast (Figure 1).
  a. If parasites are motile and in rafts, the medium is considered optimal. The starter culture can be used to begin the bulk growth for LPG production.
  b. If parasites have not formed rafts, incubate for 2 more days at 26°C in a non-shaking incubator.
    i. Check culture microscopically. When parasites are in rafts and motile, proceed with step 7a.
    ii. If parasites remain as singlets, incubate for 2 more days.
    iii. If the culture remains non-motile and as singlets after 7 days in culture, a fresh culture has to be started with a different batch of FBS.

Methods video S1. Procyclic promastigotes in logarithmic growth phase, related to step 7

## Key resources table

**Table:** 

REAGENT or RESOURCE	SOURCE	IDENTIFIER
**Biological samples**		

Heat-inactivated FBS, origin Brazil	Gibco	CAT # 10500-064

**Chemicals, peptides, and recombinant proteins**		

DMSO	Sigma-Aldrich	CAT # 472301
Protease inhibitor cocktail	Sigma-Aldrich	CAT # P8340
Ethanol ≥99.9%	Merck	CAT # 1.00983.1000
Methanol	Merck	CAT # 1.06009.1000
Diethyl ether	Merck	CAT # 1.00921.1000
Pyridine	Sigma-Aldrich	CAT # 1097280100
Ammonium hydroxide (NH_4_OH) 25%	Sigma-Aldrich	CAT # 1054280250
Chloroform	Merck	CAT # 1.02445.1000
Magnesium chloride (MgCl_2_)	Sigma-Aldrich	CAT # 8147330500
Acetic acid (C_2_H_4_O_2_)	Sigma-Aldrich	CAT # 695092
Sodium chloride (NaCl)	Merck	CAT # 1.06404.1000
Di-chloromethane	Sigma-Aldrich	CAT # 270997
Triethylamine	Sigma-Aldrich	CAT # 90335
Sulfuric acid	Merck	CAT # 1.00731.1000
Paraformaldehyde	Merck	CAT # 1.04003.1000

**Critical commercial assays**		

Pierce BCA protein assay kit with dilution-free BSA protein standards	Thermo Fisher Scientific	CAT # A55864

**Experimental models: Organisms/strains**		

*Leishmania* (*Viannia*) *braziliensis*, strain IMG3	Clinical isolate/IPTSP-UFG	N/A
*L.* (*L.*) *chagasi*, strain PP75	Clinical isolate/IPTSP-UFG	N/A
*L.* (*L.*) *amazonensis*, strain PH8	Clinical isolate/IPTSP-UFG	N/A
*L.* (*L.*) *guyanensis*, strain M4147, strain PLR6	Clinical isolate/IPTSP-UFG	N/A

**Other**		

Phosphate-buffered saline (1×) without calcium and magnesium	Lonza	CAT# BE17-516F
Grace’s insect medium (1×) non-supplemented	Gibco	CAT # 11595030
Glutamine supplement	Life Technologies	CAT # 35050087
Penicillin/streptomycin	Life Technologies	CAT # 15140122
SepaFlash column iLOK series empty solid load cartridge 4G	Screening Devices	CAT # SD-0000-004
Phenyl-Sepharose CL-4B	Merck	CAT # 17-0810-01
TLC silica gel 60 F254	Sigma-Aldrich	CAT # 1058080001
Glass Pasteur pipettes	VWR	CAT # 612-3813
24-well clear flat-bottom TC-treated cell culture plate	Corning	CAT # 353047
Inverted microscope for cell culture, 10x objective	N/A	N/A
Hemocytometer	N/A	N/A
5 mL syringe	Becton Dickinson	CAT # 307731
Screw cap tube, 15 mL	Sarstedt	CAT # 62554502
Cryovials	Greiner	CAT #123263
Microplate reader BioTek 800 TS	BioSPX	N/A

## Materials and equipment

**Table:** *Leishmania* culture medium

Reagent	Final concentration	Amount
Grace’s Insect Medium	N/A	390 mL
FBS	20%	100 mL
Glutamine supplement	1000×	5 mL
Penicillin/streptomycin	1000×	5 mL
**Total**	**N/A**	**500 mL**

Store at 4°C for up to 3 months.

**Table:** Freezing medium

Reagent	Final concentration	Amount
FBS	90%	5.4 mL
DMSO	10%	0.6 mL
**Total**	**N/A**	**6 mL**

Store on ice and use fresh.

**CRITICAL:** DMSO can penetrate the skin. Wear gloves and avoid skin contact.

**Table:** Buffer A

Reagent	Final concentration	Amount
Chloroform	3 parts	15 mL
Methanol	2 parts	16.7 mL
MilliQ	1 part	8.3 mL
**Total**	**N/A**	**50 mL**

Store in tightly closed glass containers away from light at 21°C for up to one week.

**CRITICAL:** Chloroform and methanol are toxic. Work in a fume hood, and use gloves, eye protection, and a lab coat. Avoid inhalation, skin contact, and prolonged exposure.

**Table:** Buffer B

Reagent	Final concentration	Amount
MgCl_2_	4 mM	19.04 mg
MilliQ	N/A	50 mL
**Total**	**N/A**	**50 mL**

Store tightly closed at 21°C for up to three months.

**Table:** Buffer C

Reagent	Final concentration	Amount
Chloroform	1 part	21.7 mL
Methanol	1 part	21.7 mL
MilliQ	0.3 parts	6.6 mL
**Total**	**N/A**	**50 mL**

Store in tightly closed glass containers at 21°C for up to one week.

**CRITICAL:** Chloroform and methanol are toxic. Work in a fume hood, and use gloves, eye protection, and a lab coat. Avoid inhalation, skin contact, and prolonged exposure.

**Table:** Buffer E

Reagent	Final concentration	Amount
MilliQ	15 parts	20.8 mL
Ethanol	15 parts	20.8 mL
Diethyl ether	5 parts	6.9 mL
Pyridine	1 part	1.3 mL
NH_4_OH 25%	0.017 parts	0.02 mL
**Total**	**N/A**	**50 mL**

Store in tightly closed glass containers at 21°C in the dark for up to one week.

**CRITICAL:** Ethanol and diethyl ether are highly flammable. Keep away from flames. Diethyl ether can form explosive peroxides upon exposure to air and light. Handle in a fume hood. Pyridine is highly toxic and flammable. Work in a fume hood, avoid inhalation and skin contact. Ammonium Hydroxide can cause skin irritation. Use gloves, eye protection, and a lab coat when working with buffer E, and avoid skin contact.

**Table:** Buffer L

Reagent	Final concentration	Amount
C_2_H_4_O_2_	0.1 M	30.03 g
NaCl	0.1 M	29.22 g
MilliQ	N/A	50 mL
**Total**	**N/A**	**50 mL**

Store in tightly closed glass containers at 21°C for up to three months.

**CRITICAL:** Acetic acid is corrosive and can cause burns. Use gloves, eye protection, and a lab coat.

## Step-by-step method details

### Growing of Leishmania parasites

**Timing: Approximately 4 weeks; variable**

This section describes the procedures of expanding *Leishmania* cultures *in vitro* and growing large amounts of parasites in different lifecycle stages.

1. Prepare the *Leishmania* culture medium.
2. Thaw procyclic parasites (10 × 10^6^) in 37°C. Alternatively, use the culture from the medium optimization and proceed with step 8.
3. Add 6 mL medium to a 15 mL falcon tube and add the cells drop-wisely while continuously gently mixing.
4. Centrifuge for 10 min at 3000 × *g* at 21°C.
5. Resuspend the parasites in 1 mL medium and seed to a 24-well polystyrene cell culture plate (1 mL/well).
6. Culture the parasites at 26°C in a non-shaking incubator for 2 days.

***Note:*** Close the plate with plastic tape to avoid evaporation of the culture medium.

**CRUCIAL:** The procyclic promastigotes growth best in a small medium cultured in a 24-well plate.

7. Check culture microscopically in the culture plate to determine whether parasites have adjusted to the medium.

***Note:*** Adjusted parasites will be motile and form rafts (Methods video S1) (problem 1), and the majority of the culture is in the insect stage (flagellated form). Movement of the flagellum is visible under a light microscope using phase-contrast (Figure 1). If parasites remain non-motile and as singlets after 7 days in culture, it is advisable to start with a fresh culture.

8. Expand the culture.
  a. Mix the pro-cyclic promastigotes well using a 1 mL pipette to disrupt rafts.
  b. Collect all parasites in a 15 mL tube and centrifuge for 10 min at 3000 × *g* at 21°C.
  c. Resuspend in 1 mL medium and count flagellated promastigotes. Procyclic promastigotes can be recognized by their flagellum and make up most of the culture. Non-flagellated parasites are differentiated and should not be counted (Figure 1).
    ***Note:*** To count parasites, dilute the parasites 1:10 in paraformaldehyde 0.4% and count them using a hemocytometer.
  d. Adjust the concentration to 1 × 10^6^/mL with medium and expand maximally in a taped 24-well plate (1 mL/well) to avoid evaporation.
  e. Culture the parasites at 26°C for 2–3 days in their exponential growth phase (problem 2).
    ***Note:*** The growth is considered exponential when cultures expand 5–10× every 2 days.
9. Maintain the culture of procyclic promastigotes (logarithmic phase parasites).
  **Crucial:** Parasite cultures need to be split every 2–3 days to maintain parasites in the procyclic promastigote (logarithmic) growth phase.
  a. Determine parasite numbers as in step 8.
  b. Collect parasites in cryovials in PBS. No minimum or maximum amount of parasites is required for this step. Store parasites at −20°C for LPG extraction. Snap freezing is not necessary.
  c. Expand remaining parasites in culture (1 × 10^6^/mL) for the experimental requirements and continue the culture as described in step 9.
  Methods video S2. Metacyclic promastigotes in stationary growth phase, related to step 10
10. Optionally, differentiate the culture to metacyclic promastigotes (stationary phase parasites).
  a. Expand the culture maximally as described in step 8.
  b. Incubate the culture for 6–7 days at 26°C without refreshing the medium. Parasites will be motile and flagellated but not in rafts anymore in the stationary growth phase (Methods video S2).
  c. After 6–7 days metacyclic promastigotes can be harvested from the medium as described in step 9 for LPG extraction. Snap freezing is not necessary.

### Generating parasite lysates

**Timing: 1 h**

In this section, we describe how lysates are generated from fresh *in vitro* cultures and stored until LPG extraction.

11. Mix logarithmic- or stationary-phase parasites well with a 1 mL pipette and collect all medium in a 50 mL falcon tube.
12. Double the collected volume 1:1 using PBS.
13. Centrifuge for 10 min at 3000 × *g* at 21°C, remove the supernatant, and resuspend in 1 mL PBS.
14. Repeat the washing step twice.
15. Determine parasite numbers as in step 8.
16. Add protease inhibitors 1:100.
17. Disrupt the parasite cell membrane to generate lysates by six freeze/thaw cycles in liquid nitrogen and a 37°C water bath.

***Optional:*** Perform protein quantification using a BCA protein assay to determine protein content.

### Freezing new starter cultures

**Timing: 1 h**

This section describes how to freeze fresh starter cultures from procyclic promastigotes to ensure that cultures can be kept at a low passage number. For this step, prepare freezing medium on ice and pre-cool a freezing container to 4°C at least 2 h before starting.

18. Determine parasite numbers from a procyclic, exponentially growing culture with low passage number as in step 8.
19. Adjust the concentration to 8 × 10^6^/mL in freezing medium and transfer 1.5 mL cells/cryovial.

***Note:*** Keep cryovials on ice and work fast, as DMSO is toxic for the cells.

20. Transfer vials to the freezing container, and freeze cells at −80°C for at least 24 h. Cells must be transferred to liquid nitrogen within 7 days for long-term storage.
21. Keep a sufficient amount of parasites in culture (1 × 10^6^/mL) for the experimental requirements and continue the culture as described in step 9.

***Note:*** Parasites should ideally not be used after 10 passages, as they lose their virulence (problem 3).

***Note:*** Expand culture maximally to freeze down sufficient vials of a lesion isolate to allow a restart of the culture after maximally 15 passages.

### Lipophosphoglycan extraction

**Timing: Approximately 2 days**

LPG is first extracted from the membrane of Leishmania lysates using apolar mixtures (buffer A, B, and C) that dissolve lipids readily. Buffer A dissolves the outer membrane, and then lipids dissolve in the organic (chloroform) layer. Buffer B washes out small molecules and salts from the cytoplasm after cells have been fixed and permeabilized with buffer A. Buffer C has the same function as buffer A and is used as a purification step. Buffer E contains pyridine which will ensure that the phosphor groups are deprotected, binding pyridine, helping the LPG to stay in solution.

**CRITICAL:** All extraction steps are performed by resuspending pellet in the appropriate buffer and 5 min incubation at 4°C, followed by centrifugation at 3000 × *g* at 21°C for 5 min.

22. Prepare buffers A, B, C, and solvent E.

***Note:*** Buffers are volatile. Ensure working in a fume hood with adequate glass ware and always close buffers after use to avoid evaporation of alcohols.

23. Pool lysates in 15 mL tubes.

***Note:*** Do not pool procyclic and metacyclic promastigotes as well as lysates from different strains and species.

***Note:*** Divide pooled lysates across tubes to an approximate concentration of 25∗10^9^ parasites/tube.

***Note:*** If different parasite numbers are used, buffer volumes need to be adjusted accordingly.

24. Pellet the lysates for 5 min at 3000 × g at 21°C.
25. Resuspend the pellet in 7.5 mL of buffer A.
  a. Mix buffer A on a magnetic stirrer continuously to avoid separation of chloroform from the aqueous phases.
    ***Note:*** Stir bar must be coated in PTFE (polytetrafluoroethylene) (Methods video S3).
  b. Use a glass pipette and shake vigorously to dissolve pellet.
  c. Incubate the suspension for 5 min at 4°C, followed by centrifugation at 3000 × *g* at 21°C for 5 min.
  d. Discard the supernatants and repeat step 26 once. Then move to step 27.
    ***Note:*** Buffer A forms a bi-layer with the pellet in between both layers. To discard the supernatant, decant the top layer. Then tap the tube gently to move the pellet and remove the bottom layer with a glass Pasteur pipette (Methods video S4).
26. Resuspend the pellet in 7.5 mL of buffer B.
  a. Use a spatula and shake vigorously to dissolve pellet.
  b. Discard the supernatants and repeat step 27 three more times. Then move to step 28.
27. Resuspend the pellet in 7.5 mL of buffer C.
  a. Use a glass pipette and shake vigorously to dissolve pellet. Ensure homogenous mixing of buffer C as described in step 26a (Methods video S3).
  b. Discard the supernatants and repeat step 28 two more times. Then move to step 29.

***Note:*** Buffer C forms a bi-layer with the pellet in between both layers. Carefully decant the top and bottom layer together. The pellet sits loose in the tube.

28. Resuspend the pellet in 7.5 mL of solvent E.
  a. Use a spatula and shake vigorously to dissolve pellet.
  b. Collect the supernatants in a clean glass bottle.
  c. Repeat step 29 three more times and discard the pellet after the 4th extraction step.
29. Lyophilizate the supernatants of solvent E under vacuum in 15 mL falcon tubes to obtain raw LPG.

Methods video S3. Pellet resuspension in buffer A and buffer C, related to steps 26 and 28

Methods video S4. Discarding of buffer A and buffer C bi-layers, related to steps 26 and 28

### Lipophosphoglycan purification

**Timing: Approximately 2 days**

These steps describe the purification of *Leishmania* LPG from freeze-dried raw LPG. Buffer L is used as hydrophilic aqueous solvent that is compatible with the used Sepharose columns. Acetic acid facilitates protein precipitation and aids elution from the Sepharose gel.

***Note:*** 10^9^ metacyclic parasites (*L. braziliensis*) yield approximately 1 mg lysates or 0.25 mg LPG. Yields differ between lifecycle stages, species, and strains (problem 3).

**Figure 2:**
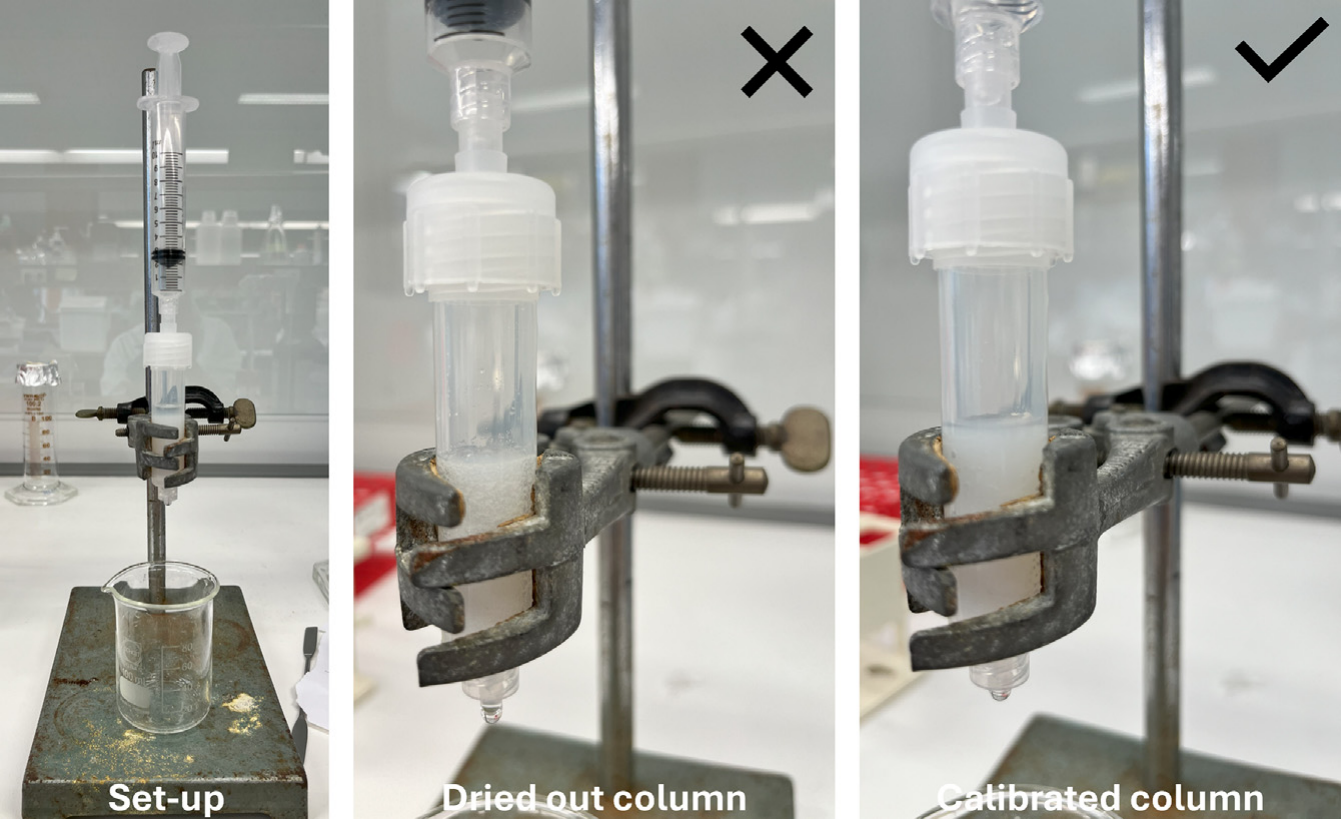
Setup and preparation of Sephadex G015 columns (Left) column set-up with syringe to add phenyl-Sepharose, buffer L, or air to the column and a beaker to collect flowthrough; (middle) drying out the column should be avoided; (right) prepared column with phenyl-Sepharose and buffer L.
31. Resuspend lyophilized LPG in buffer L (2 mL/column).
  a. Flush the collection tubes to remove all the LPG.
  b. Pool the fractions.
32. Add LPG suspension to the column.
  a. Flush sample tube with 1 mL buffer L.
  b. Add wash fraction onto the column.
33. Wash column with buffer L (10 mL).
34. Wash column with 0.1 M acetic acid (4 mL).
35. Wash column with MilliQ (4 mL).
36. Elute LPG with buffer E (10 mL) and collect the flow through in a clean glass bottle.

***Note:*** Weigh the containers in which you freeze-dry your samples with an exact balance prior to collecting the LPG containing fraction in your sample. Weigh the containers after freeze-drying again to determine the difference in weight. The difference is the amount of isolated LPG.

30. Prepare and calibrate Sephadex G-15 columns (Figure 2).
  ***Note:*** Approximately 5.5 mL phenyl-Sepharose/50∗10^9^ parasites are required. Estimate the amount of needed columns accordingly.
  a. Add 5.5 mL phenyl-Sepharose/column using a syringe without drying the column out (Figure 1).
  b. Remove bubbles with tapping against the column.
  c. Equilibrate the column with buffer L by adding 6 mL/column for three times using a syringe.
  d. Add 5.5 mL phenyl-Sepharose/column using a syringe without drying the column out (Figure 1).
  e. Remove bubbles with tapping against the column.
  f. Equilibrate the column with buffer L by adding 6 mL/column for three times using a syringe without drying the column out to avoid air bubbles (Figure 2).
  g. Repeat step c two more times.
  h. Dry the column until just above the phenyl-Sepharose layer by adding air to the column using a syringe (Figure 2).
    ***Note:*** Columns can be re-used if stored in 25%–30% EtOH at 4°C, and properly washed and equilibrated before use.

***Optional:*** Determine the presence of sugar molecules with thin layer chromatography (TLC) (problem 4).

37. Dry LPG containing fraction under N2/Argon evaporation.

***Note:*** There are volatile solvents in the elutes (Ethanol and diethyl ether) with low freezing- and boiling points which are preferably removed before freeze-drying (or freezing at −80 degree until freeze-drying).

38. Lyophilizate the samples under vacuum in 15 mL falcon tubes to obtain LPG.
39. Resuspend LPG in PBS with 0.5% DMSO and perform protein quantification using a BCA protein assay to determine protein content. LPG structure and purity is not determined with this procedure but can be assessed as described elsewhere (problem 5).^1^
40. Store at −20°C. Avoid freeze-thaw cycles.

## Expected outcomes

**Figure 3:**
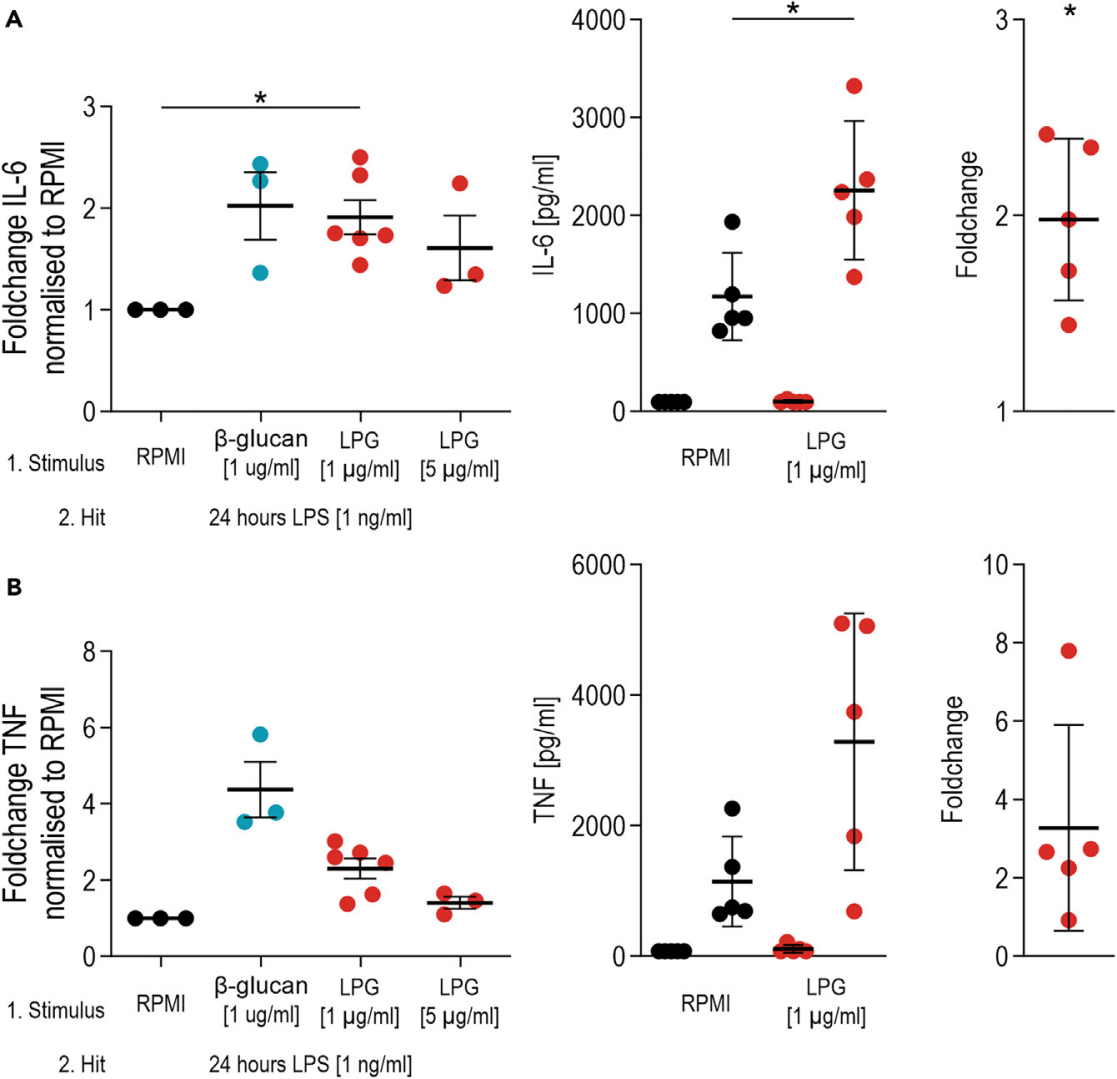
Expected cytokine production by human monocytes after the induction of trained immunity *in vitro* by LPG and secondary restimulation with LPS RPMI, control for non-trained cells; β-glucan, training stimulus control (∗ p < 0.05 by Wilcoxon test).

After the induction of trained immunity in adherent monocytes,^6^ the cells that have been exposed to LPG for a brief period will present with increased production of IL-6 and TNF in response to LPS stimulation for 24 h (Figure 3). Training responses vary between donors.

## Quantification and statistical analysis

Thin layer chromatography (TLC) can be used to determine whether the LPG extraction and purification yielded in a product containing sugars. This is not a structural analyses but rather a check whether the product contains a sugar molecule.

In order to prevent contamination of the TLC plates, wash your hands or use gloves while cutting to prevent and touch the silica side of the plates as least as possible.

TLC plates are inactivated by running them in 10 mL of a mixture of 5% triethylamine (TEA) in dichloromethane (DCM). They are dried to air under nitrogen flow or in vacuo prior to further use. Using a glass pipette, a small spot of the sample is applied to a plate, about 1 cm from the bottom edge. Spot 5 times on the same spot while letting the spot dry in between spotting. It is essential that the plate is completely dry before adding eluent.

The plate is developed in TCL eluent. TCL eluent is poured into a glass container wide enough to fit the TCL plate to a depth of less than 1 cm. The TLC plate is placed in the chamber so that the spot(s) of the sample are just above the eluent surface, and the lid is closed. The plate is removed when the solvent has soaked 90% of the plate. The plate is dried to air, under nitrogen flow or in vacuo, and stained by dipping the plate into 10% sulfuric acid in methanol. The 10% sulfuric acid is prepared by carefully adding concentrated sulfuric acid to methanol on ice.

Char the dried plates using a heat-gun. LPG is visible as a faint brown spot compared to the white silica of the TLC plate.

## Limitations

One other study has described a similar protocol to extract LPG from Leishmania parasites (https://doi.org/10.1371/journal.pntd.0004848). The advantage of this technique over older extraction protocols is that no radioactive labeling is required. Disadvantage of this approach is that it is a time intensive protocol, as large amounts of parasites are required for LPG generation. This is a limitation, since the parasites do not grow in big volumes in the same or even higher concentration.

FBS-sensitive species may not grow in FBS available in all countries (we have observed differences in FBS obtained from Brazil to the Netherlands, which may be attributed to the endogenous regions of the parasites).

The isolation and purification of LPG using this protocol is validated,^1^ however the exact structure of the obtained macromolecule is not determined with the here presented methods.

## Troubleshooting

### Problem 1

The starting culture develops unexpectedly (Point 7).

### Potential solutions

There are multiple reasons why a starting culture can behave unexpectedly.

• The parasites may have been stored too long or been frozen inadequately. A non-growing culture due to this reason is hard to recover without exceeding a passage number of 15. Best is to start a new culture with fresh medium.
• The culture has been thawed incorrectly. When starting the culture, it is important to perform the steps very slowly and with warm medium, as the parasites are very sensitive to medium changes. Importantly, when starting from a frozen culture, the passage number is not zero but “passage number of the frozen culture + 1”.
• The starting culture may have contained more/less parasites due to a count error. When there are too many parasites in culture, stationary phase will be reached earlier, leading to an increase of dead parasites on the day of harvest. Conversely, a lower number of parasites in the starting culture can cause the culture to die prematurely, because stationary phase cannot be reached. Starting a new culture is advised. If the problem persists or when working with a new species/strain, performing a growth curve can be useful.^7^ Usually, higher numbers of metacyclic parasites are observed two to three days after the beginning of the stationary phase.

### Problem 2

The parasites do not grow exponentially (Point 8e).

### Potential solution

Change of medium using a different FBS batch may recover the culture. Some species are very sensitive to variation in the nutrients. If the problem persists, it is best to start a new culture considering the points of problem 1.

### Problem 3

The LPG yield is lower than expected (Point 22 & lipophosphoglycan purification).

### Potential solutions

• The parasites were in culture for too long, leading to a loss of virulence. It is best not to exceed 10 passages when using *Leishmania* for immunological assays or LPG extraction. Alternatively, infecting mice can recover virulence.
• Other possible reasons for a low yield can be the life cycle stage (metacyclic promastigotes have more LPG than procyclic promastigotes) or the *Leishmania* species or strain. These differences are to be expected and are not necessarily due to an error in the extraction or purification.

### Problem 4

There is no LPG visible in the TLC (Point 37).

### Potential solution

Possibly, the LPG has eluted in one of the wash steps. Collect all fractions (buffer L, acetic acid, MilliQ, and solvent E) separately to check whether LPG has eluted in the wrong fraction.

The LPG stain after charring can be very faint when the input amount of parasites was low. Functional validation of extraction and purification is still advised.

### Problem 5

Unexpected structures show up in the LPG isolate by NMR spectroscopy (Point 40).

### Potential solution

It is likely that the lysates were contaminated at one of the extraction and purification steps. It is crucial to handle all cultures sterile during parasite growth, collection, and lysate generation.

While extraction and purification are not performed under sterile conditions, a clean workplace is essential. Thorough cleaning of the fume hood used for these steps is advised. When re-using phenyl-Sepharose and/or columns ensure that these were properly stored in 25%–30% EtOH at 4°C, washed, and equilibrated before use.

## Resource availability

### Lead contact

Further information and requests for resources and reagents should be directed to and will be fulfilled by the lead contact, Jéssica C. dos Santos (Jessica.DosSantos@radboudumc.nl).

### Technical contact

Technical questions on executing this protocol should be directed to and will be answered by the technical contact, Lisa Teufel (Lisa.Teufel@radboudumc.nl).

### Materials availability

This protocol does not generate new unique reagents.

### Data and code availability

This protocol does not generate or analyze any datasets or codes.

## Acknowledgments

We thank Peter Moons and Emiel Rossing (Radboud University, Nijmegen, the Netherlands) for their technical help and Prof. David Williams (East Tennessee State University, Johnson City, TN, United States) for his effort in characterizing the purification product. We thank Prof. Leo Joosten (Radboudumc, Nijmegen, the Netherlands) for his scientific support. We thank Prof. Dr. Milton Adriano Pelli de Oliveira (Universidade Federal de Goiás, Goiânia, Brazil) for providing videos and images of the *in vitro* cultures.

## Author contributions

All the authors helped to conceive and/or optimize the protocols. L.U.T. wrote the manuscript, and J.C.d.S. read the manuscript and supervised the work.

## Declaration of interests

The authors declare no competing interests.
